# Psychosocial working conditions and sickness absence among younger employees in Denmark: a register-based cohort study using job exposure matrices

**DOI:** 10.5271/sjweh.4083

**Published:** 2023-05-01

**Authors:** Jeppe K Sørensen, Jacob Pedersen, Hermann Burr, Anders Holm, Tea Lallukka, Thomas Lund, Maria Melchior, Naja H Rod, Reiner Rugulies, Børge Sivertsen, Stephen Stansfeld, Karl B Christensen, Ida EH Madsen

**Affiliations:** 1 National Research Centre for the Working Environment, Copenhagen, Denmark.; 2 Unit Psychosocial factors and mental health, Department of Work and Health, Federal Institute for Occupational Safety and Health (BAuA), Berlin, Germany.; 3 Department of Sociology, Western University, Canada.; 4 Department of Public Health, University of Helsinki, Helsinki, Finland.; 5 Department of Occupational and Social Medicine, Holbæk Hospital, Holbæk, Denmark.; 6 Section of Social Medicine, Department of Public Health, University of Copenhagen, Copenhagen, Denmark.; 7 Sorbonne Université, INSERM, Institut Pierre Louis d’Épidémiologie et de Santé Publique (IPLESP), Equipe de Recherche en Epidémiologie Sociale (ERES), Paris, France.; 8 Section of Epidemiology, Department of Public Health, University of Copenhagen, Copenhagen, Denmark.; 9 Department of Public Health, University of Copenhagen, Copenhagen, Denmark.; 10 Department of Health Promotion, Norwegian Institute of Public Health, Bergen, Norway.; 11 Department of Research & Innovation, Helse Fonna HF, Haugesund, Norway.; 12 Department of Mental Health, Norwegian University of Science and Technology, Trondheim, Norway.; 13 Centre for Psychiatry, Barts and the London School of Medicine, Queen Mary University of London, London, UK.; 14 Section of Biostatistics, Department of Public Health, University of Copenhagen, Copenhagen, Denmark.

**Keywords:** JEM, labor market entry, multi-level analysis, Poisson regression, register follow-up, sick leave

## Abstract

**Objective:**

Previous literature has established associations between psychosocial working conditions and sickness absence (SA), but only few studies have examined associations among younger employees. This study aimed to investigate associations between psychosocial working conditions and SA among employees, aged 15–30 years, who entered the labor market in Denmark between 2010 and 2018.

**Method:**

We followed 301 185 younger employees in registers for on average 2.6 years. Using job exposure matrices, we assessed job insecurity, quantitative demands, decision authority, job strain, emotional demands, and work-related physical violence. Adjusted rate ratios of SA spells of any length were estimated for women and men separately with Poisson models.

**Results:**

Among women, employment in occupations with high quantitative demands, low decision authority, high job strain, high emotional demands, or high work-related physical violence was associated with higher rates of SA. Being employed in occupations with high versus low emotional demands showed the strongest association with SA, with a rate ratio of 1.44 [95% confidence interval (CI) 1.41–1.47]. Among men, being employed in occupations with low decision authority showed the strongest association with SA (1.34, 95% CI 1.31–1.37), whereas occupations with high quantitative demands, high job strain, and high emotional demands were associated with lower rates of SA.

**Conclusion:**

We found that several psychosocial working conditions were associated with SA spells of any length. Associations with SA spells of any length resemble associations with long-term SA, suggesting that results from previous studies on long-term SA may be generalizable to all lengths of SA among younger employees.

Sickness absence (SA) is a major public health concern (1, 2). SA is associated with an increased risk of labor market exclusion (3) and is of great importance for both those directly affected and their employers. Average rates of SA vary between 3% and 6% in Europe, and it is estimated that the cost of SA accounts for about 2.5% of GDP (4). Many different factors may affect the risk of SA including psychosocial working conditions. Previous studies linking psychosocial working conditions with SA have shown associations with job control (5–11), psychological and emotional demands (5, 9–12), job strain (8, 13, 14), workplace violence (10, 15), and job insecurity (16).

However, previous research has mainly focused on the whole working population (5–10, 12, 13, 15–17), and only a few studies have examined SA among younger employees (11, 14). Additionally, many previous studies have focused on the risk of long-term SA (5, 8, 10, 12–15) and fewer studies have focused on all lengths of SA (6, 7, 9, 11). This is an important gap in the existing knowledge for the following reasons: (i) Younger employees have different SA patterns compared to older employees with shorter spells of SA being more common (18, 19). Age may be an important effect modifier of the association between work environment and SA (11, 14, 20), and consequently results from previous studies on older populations may be less generalizable to younger populations. (ii) A study of younger employees in Denmark has found accumulation of low job control associated with a higher risk of disability pension (21). As younger employees are expected to have longer lifetime exposure to occupational hazards, establishing associations between working conditions and labor market affiliation in the beginning of the work-life may be particularly important. (iii) Higher rates of SA among young employees may be a risk marker of more permanent exclusion from the labor market (3, 22). Early exclusion from the labor market may not only affect the individual but may also have societal consequences in terms of lost productivity and payment of social benefits. Hence, identification of work-related risk factors for SA in younger employees may be considered particularly prudent and knowledge of potentially modifiable risk factors may be used to prevent SA among younger employees at the beginning of their working careers.

This study aimed to investigate associations between psychosocial working conditions and SA among younger employees in Denmark using a register-based cohort of 301 185 young employees. SA was recorded on a daily basis through company registers and psychosocial working conditions were measured as job insecurity, quantitative demands, decision authority, job strain, emotional demands, and work-related physical violence on occupational level. These six psychosocial working conditions have been associated with SA in previous studies (5–13, 15, 16), and we know from previous validation that these factors reasonably can be estimated on an occupational level (23). To prevent post hoc decision-making, we published a study protocol (https://doi.org/10.6084/m9.figshare.19501495.v1) containing detailed descriptions of the hypothesis, design, and methods (24).

## Methods

### Study design

**Figure 1:**
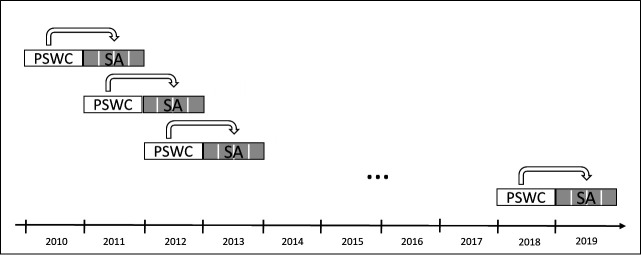
Prospective study design of how repeated measurement of psychosocial working conditions (PSWC) were related to sickness absence (SA) the following year during follow-up.

We used data from an updated version of the Danish Work Life Course Cohort study (DaWCo) with all younger employees (aged 15–30 years) who entered the Danish labor market for the first time between 2010 and 2018. Details of DaWCo have been published elsewhere (25). Briefly, DaWCo is an open nationwide cohort following individuals from labor market entry until the end of follow-up (end of 2019) in Danish registers. In DaWCo, working conditions are measured on the occupational level using job exposure matrices (JEM) (26). SA was measured with data from the Danish Register of Work Absence (RoWA), which is a combination of two registers; Statistics Denmark’s ‘Absence and Employment’ and ‘Periods of Absence’ (27). RoWA includes information on the daily absence for all public employees, all private employees in large companies (>250 employees), and a yearly representative sample of private employees from middle-sized companies (10–250 employees). To ensure a prospective design, we related repeated yearly estimated psychosocial working conditions with number of SA spells the following year. Figure 1 presents the prospective design.

### Population

In total, 579 114 individuals, aged 15–30 years, entered the Danish labor market for the first time between 2010 and 2018. We were able to follow 301 778 (52%) employees during follow-up in RoWA. We excluded individuals who emigrated (N=397) or received a disability pension (N=150) before or in the baseline year. We further excluded 46 individuals with missing information on sex. The final study population consisted of 301 185 individuals (160 104 women and 141 081 men) with 771 976 person-years at risk. Individuals were followed in registers from labor market entry until the end of 2019 with a mean follow-up time of 2.6 years [interquartile range (IQR) 1.0–3.7].

### Assessment of psychosocial working conditions

Psychosocial working conditions including job insecurity, quantitative demands, decision authority, job strain, emotional demands, and work-related physical violence were estimated yearly using JEM as time-varying exposures from 2010 until 2018. The construction and validation of the JEM are described in the supplementary material (www.sjweh.fi/article/4083), Appendix 1 and more detailed elsewhere (23). In short, the JEM are constructed based on self-reported data from the 2012 wave of the Working Environment and Health in Denmark study (WEHD) containing 17 591 respondents aged 18–64 (12% were aged 18–30 years) (28). The estimated values from the JEM were assigned yearly to each individual in DaWCo based on yearly job title (DISCO-08), age, and sex. In line with previous JEM-based studies, 18-year-old age-specific JEM were assigned to employees aged 15–17 years (29, 30). We categorized estimated values of the six psychosocial working conditions into groups based on quartiles of the yearly distribution for women and men separately. Supplementary material Appendix 1, table S2 presents the ten occupational groups with the highest average level or risk of the six psychosocial working conditions.

### Assessment of sickness absence

We registered all spells of SA repeatedly from 1 January 2011 until the end of follow-up 31 December 2019, but disregarded SA due to caring for a sick child and SA due to work-related accidents. To account for potential seasonal variation in rates of SA spells, we quantified the number of SA spells of any length (≥1 day) in three-month windows (January–March, April–June, July–September, and October–December). In Denmark, all employees are guaranteed SA benefits during illness. During the first 30 days, benefits are paid by the employee, after which benefits are paid by the municipality. Benefits may vary according to agreements made by different trade unions (31).

### Assessment of covariates

We included a range of register-based covariates. Supplementary material appendix 2 table S3 presents an overview of categorizations and sources. We included the following sociodemographic covariates: age, migration background (no migration background, immigrant, or descendent of immigrants), and cohabitation. To account for cohort effects, we included calendar year, years of labor market entry, years since labor market entry, years with employment, and sector of employment (public or private). To account for socioeconomic position, we included personal disposable income. We included information on annual health services used and information on hospital-diagnosed chronic somatic disorders (type-2 diabetes, coronary heart disease, stroke, cancer, asthma, chronic obstructive pulmonary disease) and mental disorders (ICD-10 psychiatric diagnoses F01-F99) before labor market entry. Additionally, we included a JEM on physical work demands (23). Lastly, as previous SA might affect current working conditions and risk of future SA (32), we included information on any SA spells the year before exposure, any long-term SA spells (>30 days) the year before exposure, and an indicator of >10 days of SA in the year before exposure.

For supplementary analyses, we defined four stratification variables: educational attainment [primary and lower (≤10 years of education), upper secondary and short cycle tertiary (11–15 years), and bachelor’s or higher (≥15 years)], industry (wholesale and retail trade; human health and social work; accommodation and food service activities; education; travel agent; cleaning and other operational services; public administration and defense compulsory social security; manufacturing; construction; other industries; and unknown), sector of employment (public or private sector), and age groups (15–19, 20–24, and ≥25 years).

### Statistical analyses

First, we calculated the rate of SA spells of any length (≥1 day) per person-year to assess crude associations for the six psychosocial working conditions. Second, using a multilevel Poisson regression model, we estimated adjusted rate ratio (RR) and 95% confidence interval (CI) for the six psychosocial working conditions and SA spells of any length separately. To ensure a longitudinal design we related yearly assessed psychosocial working conditions with SA spells the year after (see figure 1). During follow-up, we considered periods of non-employment as time not at risk. We treated migration, disability pension, and death as absorbing states, and individuals for whom these occurred were only considered at risk until first occurrence. All analyses were conducted separately for women and men. We included a scale parameter to account for over-dispersion and used the logarithm of the time at risk as an offset variable to account for unequal follow-up time as recommended in the literature (33). The multilevel approach accounted for repeated events of SA on an individual level and repeated measurement of working conditions on an occupational level (34).

We present a minimally and a fully adjusted model. The minimally adjusted model included covariates related to the JEM (age) and the cohort design (calendar year, year of labor market entry, years since labor market entry, and years with employment). The fully adjusted model further included sociodemographic covariates (migration background, cohabitation, and sector of employment), socioeconomic position (disposable personal income), health (health services use, existing chronic and mental disorders), physical work demands, and previous SA. Step-wise adjustment are presented in supplementary material appendix 5. All covariates except sex, migration background, year of labor market entry, and existing chronic and mental disorders were included as time-varying variables.

### Supplementary analyses

In supplementary analyses, we conducted four separate analyses estimating fully adjusted RR of SA spells of >1, >3, >7, and >30 days for the six psychosocial working conditions. We conducted four stratified analyses by educational attainment, industry, sector of employment, and age group. To ensure higher generalizability between individuals, we conducted supplementary analyses excluding observations whit employment while under education.

Lastly, we conducted quantitative bias analyses to estimate the extent of misclassification of exposure derived from the use of JEM to assess working conditions. These analyses produce bias-corrected estimates under the assumption of exposure measured without misclassification. The analyses were conducted using the method described by Lash, Fox, & Fink (35) and is described and presented in detail in the supplementary material appendix 3.

## Results

Table 1 shows the time-invariant characteristics of the participants. The mean age at labor market entry was 18 (SD 3.7, IQR 16–20) years for women and 19 (SD 4.0, IQR: 16–20) years for men. The majority had no migration background (women 75.3%, men 72.9%). Having suffered from chronic somatic disorders before labor market entry was higher among men (8.0%) than women (5.7%). Few participants had multiple chronic somatic disorders (0.2% and 0.3%, respectively). Any mental disorders before labor market entry were around 7% for both women and men. Supplementary figures S1–S6 in appendix 4 illustrates trends over time since labor market entry for the time-variant covariates. In the first year of employment, most participants were employed in “wholesale and retail trade”. During follow up employment in “human health and social work” became more prevalent among women and employment in “construction” and “public administration” became more prevalent among men.

**Table 1:** Participant characteristics. [SD=standard deviation.]

		Women					Men			
		N	%	Mean	SD		N	%	Mean	SD
Participants		160 104	53.2				141 081	46.8		
Age (labor market entry)				18.4	3.7				19.0	4.0
Migration background										
	No migration background	120 580	75.3				102 817	72.9		
	Immigrant	28 590	17.9				29 256	20.7		
	Descendent	10 934	6.8				9008	6.4		
Chronic diseases before labor market entry		9172	5.7				11 283	8.0		
Comorbidity (>1)		338	0.2				488	0.3		
Mental disorders before labor market entry		11 282	7.0				10 523	7.5		

### Psychosocial working conditions and rates of sickness absence spells

Table 2 presents the number of SA spells and minimally and fully adjusted RR (95% CI) for SA spells of any length for each of the six psychosocial working conditions. Among women, during 426 068 person-years of follow-up, we identified 693 677 spells of SA of any length (on average 1.6 spells per person-years). Among men, during 345 908 person-years, we identified 446 584 spells of SA (on average 1.3 spells per person-years). The median length of SA was one day for women (IQR 1–3) and men (IQR 1–2).

**Table 2:** Rate ratio (RR) for the association between being employed in occupations with specific psychosocial working conditions and sickness absence of any length (≥1 day) among women (N=160 104) and men (N=141 108). [PY=person-years; CI=confidence interval]

			Women						Men				
			Sickness absence rates per PY		Minimally adjusted model		Fully adjusted model		Sickness absence rates per PY		Minimally adjusted model		Fully adjusted model
					RR ^a^ (95% CI)		RR ^b^ (95% CI)				RR ^a^ (95% CI)		RR ^b^ (95% CI)
Job insecurity													
	Low		1.66		1.00		1.00		1.02		1.00		1.00
	Medium-low		1.14		0.80 (0.78–0.81)		0.82 (0.81–0.84)		1.39		1.38 (1.35–1.41)		1.26 (1.24–1.29)
	Medium-high		2.02		1.25 (1.23–1.28)		1.07 (1.05–1.09)		1.22		1.29 (1.26–1.32)		1.13 (1.11–1.15)
	High		1.75		1.07 (1.05–1.08)		0.96 (0.94–0.97)		1.55		1.50 (1.46–1.53)		1.22 (1.20–1.25)
Quantitative demands													
	Low		1.39		1.00		1.00		1.47		1.00		1.00
	Medium-low		1.54		1.18 (1.16–1.20)		1.18 (1.16–1.20)		1.22		0.86 (0.84–0.88)		0.92 (0.90–0.93)
	Medium-high		1.83		1.21 (1.18–1.23)		1.16 (1.14–1.18)		1.40		0.90 (0.88–0.91)		0.88 (0.86–0.89)
	High		1.81		1.05 (1.03–1.07)		1.14 (1.12–1.16)		1.10		0.65 (0.64–0.67)		0.76 (0.74–0.78)
Decision authority													
	High		1.52		1.00		1.00		1.08		1.00		1.00
	Medium-high		1.88		1.46 (1.44–1.49)		1.21 (1.19–1.24)		1.20		1.35 (1.32–1.38)		1.17 (1.15–1.20)
	Medium-low		1.57		1.46 (1.43–1.49)		1.17 (1.15–1.20)		1.36		1.44 (1.41–1.48)		1.21 (1.18–1.24)
	Low		1.59		1.50 (1.47–1.53)		1.27 (1.25–1.30)		1.55		1.67 (1.64–1.71)		1.34 (1.31–1.37)
Job strain													
	Low		1.44		1.00		1.00		1.51		1.00		1.00
	Medium-low		1.65		1.20 (1.18–1.22)		1.03 (1.01–1.05)		1.04		0.78 (0.76–0.80)		0.88 (0.86–0.90)
	Medium-high		1.78		1.46 (1.43–1.48)		1.22 (1.20–1.24)		1.41		0.92 (0.91–0.94)		0.92 (0.90–0.94)
	High		1.70		1.33 (1.30–1.35)		1.16 (1.14–1.18)		1.24		0.84 (0.82–0.85)		0.87 (0.86–0.89)
Emotional demands													
	Low		1.34		1.00		1.00		1.43		1.00		1.00
	Medium-low		1.37		1.08 (1.06–1.10)		1.05 (1.04–1.07)		1.25		0.91 (0.89–0.93)		1.00 (0.98–1.02)
	Medium-high		1.47		1.06 (1.04–1.08)		1.14 (1.11–1.16)		1.24		0.81 (0.79–0.83)		0.92 (0.90–0.94)
	High		2.40		1.64 (1.61–1.67)		1.44 (1.41–1.47)		1.26		0.79 (0.77–0.81)		0.97 (0.95–0.99)
Work-related physical violence													
	Low		1.42		1.00		1.00		1.35		1.00		1.00
	Medium-low		1.45		0.86 (0.84–0.87)		0.97 (0.95–0.99)		1.41		1.25 (1.23–1.28)		1.09 (1.07–1.11)
	Medium-high		1.36		0.88 (0.87–0.90)		0.93 (0.91–0.95)		1.07		0.99 (0.97–1.01)		1.04 (1.02–1.06)
	High		2.34		1.43 (1.41–1.46)		1.29 (1.27–1.32)		1.37		1.13 (1.10–1.15)		1.10 (1.08–1.12)

^a^ Minimally adjusted RR adjusted for age, calendar year, year of labor market entry, years since labor market entry, and years with employment. ^b^ Fully adjusted RR further adjusted for migration background, cohabitation, sector of employment, disposable personal income, health services use, existing chronic somatic and mental disorders, physical work demands, and previous SA.

**Figure 2:**
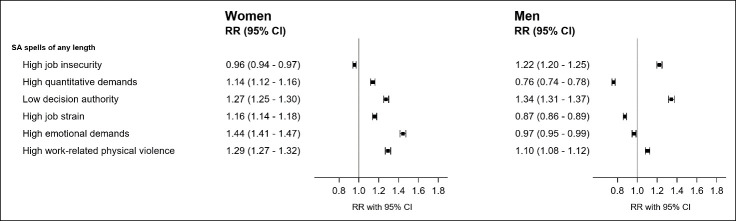
Fully adjusted rate ratio (RR) for the associations between being employed in occupations with specific psychosocial working conditions and sickness absence (SA) spells of any length (≥1 day) among women (N=160 104) and men (N=141 081). RR adjusted for age, migration background, cohabitation, sector of employment, labor market entry, years since labor market entry, years with employment, disposable personal income, health services use, exiting chronic somatic and mental disorders, physical work demands, and previous SA. [CI=confidence interval.]

Figure 2 presents fully adjusted RR for SA spells of any length among employees in occupations with high levels of the six psychosocial working conditions compared to employees in jobs with low levels of exposure. Among women higher rates of SA were found among employees in jobs with high quantitative demands, low decision authority, high job strain, high emotional demands, and high work-related physical violence. RR ranged from 1.14 (95% CI 1.12–1.16) for high quantitative demands to 1.44 (95% CI 1.41–1.47) for high emotional demands. We found slightly lower rates of SA among women in occupations with high job insecurity with a RR of 0.96 (95% CI 0.94–0.97) (figure 2). Among men, we found a different pattern, with higher rates of SA among employees who worked in jobs with high job insecurity, low decision authority, and high work-related physical violence and lower rates of SA among employees who worked in jobs with high quantitative demands, high job strain, and high emotional demands. Among men, the strongest association was found for low decision authority with a RR of 1.34 (95% CI 1.31–1.37) (figure 2). Adjustment for covariates attenuated most associations. Among women, adjustment changed the direction of the association for high job insecurity (RR 1.07 to 0.96) and strength the association for high quantitative demands (RR 1.05 to 1.14) (table 2).

### Supplementary analyses

Overall, we found similar associations between the six psychosocial working conditions and SA spells of any length, SA spells of >1, >3, >7, and >30 days (supplementary material appendix 6, figure S7). The most pronounced difference was among men, where we found that high emotional demands were associated with slightly higher rates of SA spells of >30 days with a RR of 1.11 (95% CI 1.00–1.24) compared to a RR of 0.97 (95% CI 0.95–0.99) for SA spells of any length.

In the stratified analysis, we found similar associations across educational attainment (supplementary material appendix 6, figure S8). However, among women with a bachelor’s degree, the association were strongest for low decision authority (1.57, 95% CI 1.47–1.67). Moreover, employment in occupations with high job insecurity was only associated with SA among women with low educational attainment (1.07, 95% CI 1.05–1.09). We found stronger associations in industries with a high degree of contact with patients or clients and in the public sector (supplementary material appendix 6, figure S9 and S10). Similar associations were found across age groups and when we excluded years with non-regular employment and years while under education (supplementary material appendix 6, figure S11 and 12). Quantitative bias analyses showed that bias-adjusted RR deviated further from unity when accounting for potential misclassification of exposure (supplementary material appendix 3, table S4).

## Discussion

### Summary and interpretation of main findings

In a Danish nationwide sample of 301 185 younger employees followed for 771 976 person-years, we investigated associations between occupationally assed job insecurity, quantitative demands, decision authority, job strain, emotional demands and work-place physical violence and SA spells of any length. We found that most of the examined psychosocial working conditions were associated with higher rates of SA spells of any length among women (five out of six) and half of the psychosocial working conditions among men. Lower rates of SA were found among women in occupations with high job insecurity and among men in occupations with high quantitative demands, high job strain, and high emotional demands. Overall, the patterns were similar across groups of educational attainment and age but deviated across industry and sector of employment. In the supplementary analyses, we found similar associations with SA spells of >1, >3, >7, and >30 days indicating that results from previous studies on long-term SA spells may to some extent be generalized to SA spells of any length among younger employees.

Contrary to our hypotheses, we found lower rates of SA among women in occupations with high job insecurity and among men in occupations with high quantitative demands compared to individuals in occupations with low levels of these exposures. The lower rates of SA among women in occupations with high job insecurity may be explained by less likelihood of calling in sick when actually ill, ie, presenteeism, possibly related to fear of losing the job to sickness absence. A presenteeism culture may be more prevalent in occupations with job insecurity (36) and this could explain our results. The contrary findings among men in occupations with high quantitative demands may be explained by the items used in the development of the JEM. Three out of the five items focused on work pace and two on working hours (supplementary material table S1). Scales on quantitative demands have been found sensitive to the choice of specific items (37) and items related to work pace have been found to identify more blue-collar jobs as high-demand jobs contrary to items rated to working hours. When we stratified the analyses by educational attainment, we found that employment in occupations with high level of quantitative demands was only associated with lower rates of SA among individuals with primary and secondary educational attainment but not among individuals with a bachelor degree or higher. This may suggest some misclassification of exposure across educational attainment which we did not capture sufficiently in the analyses. The job group variance for the applied JEM on quantitative demands has previously been found lower among men compared to women and has shown to be associated with a lower risk of musculoskeletal pain on the occupational level but not at the individual level (23). Together, this suggests that the results yielded by the JEM for quantitative demands among men should be interpreted with caution and that the association might have been different if working conditions was measured at individual level. We found that high levels of emotional demands were associated with higher rates of SA spells among men working in industries with a high level of contact with patients or students (eg, human health, social work, or education). This may explain the overall lower SA rates among men employed in occupations with high emotional demands, as fewer men were employed in industries with patient or student contact.

### Comparison with previous studies

Only few studies have investigated the associations between psychosocial working conditions and SA among younger employees. A Swedish twin study reported that JEM-assessed job demands were associated with a higher risk of SA of 1–30 days and SA of 31–365 days among younger employees aged 18–35 (11). A Finnish study of employees within the public sector reported a higher risk, although statistically not significant, of SA (10–365 days) for high demands and low job control (20). Despite some methodological differences between the two mentioned studies and the present study, the findings correspond.

This study adds to the existing literature by showing similar associations across SA spells of any length, SA spells of >1, >3, >7, and >30 days among younger employees. SA of different lengths have previously been investigated as mutually exclusive outcomes (5, 9) and in a recent study of Danish hospital employees, Mathisen et al (9) reported a wide range of psychosocial working conditions associated with SA of 1–3, 4–28, and >28 days with overall similar association across the three separate SA outcomes. In the present study, we refrained from analyzing short- and long-term SA as mutually exclusive outcomes because such analyses would be based on the assumption that a spell of short-term SA could not become a spell of long-term SA, which could cause selection bias (38). However, as similar patterns across different lengths of SA have been reported using different approaches, we believe this strengthens the suggestion that some of the same underlying mechanisms may explain associations between psychosocial working conditions and SA of different lengths, and hence results from previous studies on long-term SA may to some extent be generalizable to SA spells of any length among younger employees.

### Strength and limitations

This study has several strengths. This study followed more than 300 000 young individuals (aged 15–30 years) who entered the Danish labor market for the first time between 2010 and 2018 in registers including information on daily SA for all public employees and a yearly sample of private employees. Combining the cohort with JEM on working conditions provided us with a unique opportunity to focus on the association between working conditions and SA spells of any length among younger employees and to conduct relevant stratified analyses. Furthermore, we published a detailed study protocol before we carried out the analyses (24), which ensured that the analytical strategy was not affected by post-hoc decisions.

This study also has limitations. Whereas the JEM approach limits reporting bias, JEM may introduce misclassification of exposure (26). Employees working in jobs with an average high level of adverse psychosocial working conditions are not necessarily actually exposed and conversely, employees in jobs with an average low level are not necessarily actually non-exposed. The quantitative bias analyses suggested that the reported associations are likely conservative estimations of the true relations, thus the true relations may have been stronger than our estimates. The included JEM were constructed based on best linear unbiased prediction (BULP) for women and men separately in a nationwide sample of Danish employees aged 18–64 years (23). Even though age was included as a fixed effect using piecewise linear spline, we cannot reject the possibility of not capturing variation in working conditions among younger employees sufficiently and that JEM constructed only among younger employees might provide different results.

In this study, we measured daily SA for all public employees and a yearly sample of private employees from companies with ≥10 employees. Hence, generalizability to private employees from small companies might be limited. Furthermore, information on the length of SA was only available when individuals were employed which could cause misclassification of long-term spells of SA and supplementary analyses of SA spells of >30 days should be interpreted with this limitation in mind.

We did not investigate all aspects of the psychosocial work environment, but only those aspects that were suitable for analyses with a JEM. For example, we did not include social support, even though this exposure was associated with SA in previous studies (5). However, job title aggregated measurements of social support have previously shown low validity (39) and it is reasonable to assume that the level of social support may depend less on job title and instead may vary across work units.

Although we adjusted for a range of potential confounders, we were limited to data available in registers and hence we were not able to adjust for individual explanatory factors for SA such as health behaviors which might be a potential confounder or mediator of the association between working conditions and SA (40). In the fully adjusted analyses, we included information on previous SA which might increase the risk of over-adjustment as previous SA could be a consequence of previous adverse working conditions. However, stepwise adjustment revealed that previous SA for most working conditions had a small attenuating effect on the associations indicating only moderate influence from unobserved heterogeneity from previous SA. Moreover, we did not adjust the main analysis for education, because we regarded this as a substantial over-adjustment as the JEM were based on job title which is highly correlated with education. To account for socioeconomic differences we adjusted for disposable income, but some residual confounding from socioeconomic positions may still be present. To reduce the complexity of the analyses, we omitted adjustment for parental background as previous analyses in the DaWCo cohort have shown that these did not confound the association between working conditions and health (21, 29, 30).

### Concluding remarks

In a large nationwide cohort of younger employees, we found that employment in occupations characterized by high levels of several of the investigated psychosocial working conditions was associated with SA spells of any length. Associations for the specific psychosocial working conditions differed among women and men with more pronounced associations among women. Associations with SA spells of any length resemble associations with long-term spells of SA, suggesting that results from previous studies on long-term SA may to some extent be generalizable to SA spells of any length among younger employees.

## Supplementary material

Supplementary material
