# Supplementary material for: Psychosocial working conditions and sickness absence among younger employees in Denmark: a register-based cohort study using job exposure matrices
Source: Scand J Work Environ Health. 2023 May 1;49(4):249–58. doi: 10.5271/sjweh.4083 (PMC10713991; doi:10.5271/sjweh.4083)
Supplement: Supplementary material [file SJWEH-49-249-S001.pdf]

Psychosocial working conditions and sickness absence among younger employees in Denmark: a register-based cohort study using job exposure matrices[1]

by Jeppe K Sørensen, MSc,[2] Jacob Pedersen, PhD, Hermann Burr, PhD, Anders Holm, PhD, Tea Lallukka, PhD, Thomas Lund, PhD, Maria Melchior, PhD, Naja H Rod, PhD, Reiner Rugulies, PhD, Børge Sivertsen, PhD, Stephen Stansfeld, PhD, Karl B Christensen, PhD, Ida EH Madsen, PhD

1. Supplementary material
2. Correspondence to: Jeppe K Sørensen, National Research Centre for the Working Environment, Copenhagen, Denmark. [E-mail: jks@nfa.dk]

Appendix 1: The construction and validation of job exposure matrices 2
Appendix 2: Overview of covariates 6
Appendix 3: Quantitative bias analyses 7
Appendix 4: Trend over time since labour market entry 9
Appendix 5: Stepwise adjustment 10
Appendix 6: Supplementary analyses 11
References 18

Appendix 1: The construction and validation of job exposure matrices

We measured exposure to psychosocial working conditions using job exposure matrices (JEM) estimating the average sex-, age-, and occupational-specific level of job insecurity, quantitative demands, decision authority, and emotional demands, and the occupational-specific risk of job strain, and work-related physical violence. The construction and validity of the JEMs are presented elsewhere (1). Briefly, the JEMs were constructed based on self-reported exposure data from the 2012 Working Environment and Health in Denmark study (WEHD) (2). WEHD is a nationwide questionnaire-based survey on working conditions and health conducted every second year since 2012. In the 2012 wave, 17 591 employees between the age of 18 and 64 participated in the survey (response rate of 50.5%). **Table S1** presents an overview of the items and response options for the six psychosocial working conditions. Mean scales for quantitative demands, emotional demands, and decision authority were calculated if the respondents have responded to at least half of the items included for each factor with values ranging from 1 to 5. Job insecurity and work-related physical violence were dichotomized into high job insecurity ('to a very high extent' and 'to a high extent'), low job insecurity ('to some extent', 'to a low extent', and 'to a very low extent'), work-related physical violence (yes daily, weekly, monthly and less frequently), and no work-related physical violence (no, never). Job strain was defined as the combination of high quantitative demands and low decision authority by median splits of the two average scores.

Table S1 Overview of included items and response options from WEHD

Factor	Items	Response options
Quantitative demands	How often do you have time enough for your work tasks?	A
	How often do you have deadlines that are difficult to keep?	A
	How often do you get unexpected work tasks that put you under time pressure?	A
	How often are you available outside normal working hours?	A
	How often do you have to work overtime?	A
Decision authority	How often can you influence how you solve your work tasks?	A
	How often can you influence when you solve your work tasks?	A
Emotional demands	How often do you get emotional involved in your work?	A
	How often do you have to deal with problems of e.g. clients, patients, or students in your work (*not* problems of your colleagues)?	A

Job insecurity	To what degree are you worried about becoming unemployed?	B
Work-related physical violence	Have you within the past 12 months been exposed to physical violence at your workplace?	C

A: Always, Often, Sometimes, Seldom, Never. B: To a very high, extent, To a high extent, To some extent, To a low extent, To a very low extent. C: Yes, daily, Yes, weekly, Yes, monthly, Yes, less frequently, No, never

Predicted mean levels of quantitative demands, decision authority, and emotional demands were estimated with a linear mixed model using best linear unbiased predictors. The predicted mean level was estimated separately for women and men. A random intercept for job groups (DISCO-08) was included and age was included as a fixed effect using piecewise linear spline to produce job group-, sex- and age-specific JEMs. The predicted probabilities of job insecurity, job strain, and work-related physical violence were estimated using a logistic regression model including job group, sex, and age. A minimum of 10 individuals were required in each job group. The included JEMs have been validated against individual-level measurement and has be reported to predict a similar association with musculoskeletal pain as the outcome of interest (1).

Table S2 presents the top 10 job groups with the highest level of the six psychosocial working conditions for men and women, respectively, at the second level of DISCO-08. Estimated level of quantitative demands was in general high in occupations with some degree of management. Emotional demands were high in occupations within the healthcare and educational system. Among women, a low level of decision authority was more prevalent in office work, military work, and operator work. Among men, a low level of decision authority was more prevalent in occupations within rescue and surveillance, and among operators and assemblers. High risk of job strain was more prevalent among women working with rescue and surveillance, calculation and registration, and office work and among men working with food preparation, health services, and office work. High risk of job insecurity was more prevalent in women working as operators, drivers, and mobile plant operators and in men working in customer services and with craft and related trade work. High risk of work-related physical violence was more prevalent among care workers and sales workers as well as within rescue and surveillance and in the educational system.

Table S2 Top 10 job groups based on occupational assessed psychosocial working conditions among women and men

	Women	Men
**High job insecurity**		
	Assemblers	Customer Services Clerks
	Stationary Plant and Machine Operators	Handicraft and Printing Workers
	Drivers and Mobile Plant Operators	Craft and Related Trades Workers

	Laborers in Mining, Construction, Manufacturing, and Transport	Assemblers
	Other Clerical Support Workers	Cleaners and Helpers
	Hospitality, Retail, and Other Services Managers	Food Preparation Assistants
	Food Preparation Assistants	Stationary Plant and Machine Operators
	Professional Care Workers	Laborers in Mining, Construction, Manufacturing, and Transport
	Cleaners and Helpers	Street and Related Sales and Service Workers
	Information and Communications Technicians	Agricultural, Forestry, and Fishery Laborers
**High quantitative demands**		
	Hospitality, Retail, and Other Services Managers	Production and Specialized Services Managers
	Production and Specialized Services Managers	Chief Executives, Senior Officials, and Legislators
	Administrative and Commercial Managers	Administrative and Commercial Managers
	Managers	Hospitality, Retail, and Other Services Managers
	Information and Communications Technicians	Science and Engineering Professionals
	Business and Administration Professionals	Business and Administration Professionals
	Chief Executives, Senior Officials, and Legislators	Professionals
	Professionals	Business and Administration Associate Professionals
	Information and Communications Technology Professionals	Information and Communications Technicians
	Science and Engineering Professionals	Information and Communications Technology Professionals
**Low decision authority**		
	Other Clerical Support Workers	Protective Services Workers
	Armed Forces Occupations, Other Ranks	Stationary Plant and Machine Operators
	Stationary Plant and Machine Operators	Assemblers
	Handicraft and Printing Workers	Refuse Workers and Other Elementary Workers
	Customer Services Clerks	Drivers and Mobile Plant Operators
	Professional Care Workers	Other Clerical Support Workers
	Refuse Workers and Other Elementary Workers	Electrical and Electronics Trades Workers
	Protective Services Workers	Customer Services Clerks
	Drivers and Mobile Plant Operators	Food processing, woodworking, garment, and other craft and related trades workers
	Food processing, woodworking, garment, and other craft and related trades workers	Food Preparation Assistants
**High job strain**		
	Protective Services Workers	Food Preparation Assistants
	Numerical and Material Recording Clerks	Health Professionals

	Other Clerical Support Workers	Clerical Support Workers
	Health Professionals	Handicraft and Printing Workers
	Assemblers	Non-commissioned Armed Forces Officers
	Chief Executives, Senior Officials, and Legislators	Numerical and Material Recording Clerks
	Personal Service Workers	Business and Administration Associate Professionals
	Food processing, woodworking, garment, and other craft and related trades workers	Refuse Workers and Other Elementary Workers
	Technicians and Associate Professionals	Customer Services Clerks
	Electrical and Electronics Trades Workers	Sales Workers
**High emotional demands**		
	Health Professionals	Health Professionals
	Teaching Professionals	Teaching Professionals
	Professional Care Workers	Legal, Social, Cultural, and Related Associate Professionals
	Production and Specialized Services Managers	Professional Care Workers
	Service and Sales Workers	Food Preparation Assistants
	Health Associate Professionals	Chief Executives, Senior Officials, and Legislators
	Legal, Social, Cultural, and Related Associate Professionals	Health Associate Professionals
	Professionals	Hospitality, Retail, and Other Services Managers
	Managers	Legal, Social and Cultural Professionals
**Work-related physical violence**		
	Professional Care Workers	Professional Care Workers
	Service and Sales Workers	Teaching Professionals
	Protective Services Workers	Protective Services Workers
	Teaching Professionals	Health Professionals
	Health Professionals	Cleaners and Helpers
	Production and Specialized Services Managers	Personal Service Workers
	Chief Executives, Senior Officials, and Legislators	Market-oriented Skilled Agricultural Workers
	Personal Service Workers	Health Associate Professionals
	Elementary Occupations	Market-oriented skilled forestry, fishery, and hunting workers
	Agricultural, Forestry, and Fishery Laborers	Skilled Agricultural, Forestry, and Fishery Workers

Appendix 2: Overview of covariates

Table S3 Covariates, classification, and register

**Covariates**	**Classification**	**Register**
Sex	Women, men	The Danish Civil Registration system (3)
Age	Continuous	The Danish Civil Registration system (3)
Migration background	No migration background, immigrant, descendent of immigrants	The Danish Civil Registration system (3)
Cohabitation	Single, cohabited	The Danish Civil Registration system (3)
Sector of employment	Public, private	Danish Register of Work Absence (4)
Labor market entry	Categorical (2010-2018)	The Integrated Database for Labor Market Research (5)
Years since labor market entry	Categorical (1-9 years)	The Integrated Database for Labor Market Research (5)
Years with employment	Categorical (1-9 years)	The Integrated Database for Labor Market Research (5)
Annual net income	Yearly deciles	The Income Statistics Register (5)
Annual health services used	Yearly deciles	The National Health Service Register (5)
Exiting chronic somatic disorders	Hospital-diagnoses (including both main and secondary diagnoses) of one or more of the World Health Organization's priority of non-communicable chronic diseases targeted for prevention (type 2 diabetes, coronary heart disease, stroke, cancer, asthma, chronic obstructive pulmonary disease)	The National Patient Register (5)
Exiting mental disorder	Hospital-diagnoses (including both main and secondary diagnoses) of one or more of the ICD-10 psychiatric diagnoses (F01-F99)	The National Patient Register and The Psychiatric Central Research Register (5)
Physical work demands	JEM scores divided into yearly quartiles (high, medium-high, medium-low, low)	The JEM is constructed based on survey data from the WEHD study (1)
Previous sickness absence	• Any sickness absence (yes, no)<br>• Long-term sickness absence (yes, no)<br>• >10 days of sickness absence (yes, no)	Danish Register of Work Absence (4)
Education	• Primary and lower secondary (ISCED levels 1 and 2)<br>• Upper secondary (ISCED level 3)<br>• Short cycle tertiary (ISCED level 5)<br>• Bachelor or equivalent (ISCED level 6)<br>• Master (ISCED level 7)<br>• Doctoral (ISCED level 8)	Statistics Population's Education Register (5)
Industry	• Wholesale and retail trade,<br>• Human health and social work<br>• Accommodation and food service activities<br>• Education<br>• Travel agent, cleaning and other operational services<br>• Public administration and defense compulsory social security<br>• Manufacturing<br>• Construction<br>• Other industries<br>• Unknown	The Employment Classification Module (AKM) (5)

Appendix 3: Quantitative bias analyses

We conducted a quantitative bias analysis to estimate the extent of misclassification of exposure derived from the use of JEMs to assess psychosocial working conditions. The analysis produce a bias corrected estimate under the assumption that we had measured exposure without misclassification. We used the method proposed by Lash, Fox, & Fink (6) (pages 86-93). A comparison of the corrected estimates against the observed estimates gives an idea of the magnitude and direction of the expected bias as a result of misclassification of exposure. To produce the bias corrected estimates we assessed sensitivity (correctly classified as exposed) and specificity (correctly classified as non-exposed) for the six occupational assessed psychosocial working conditions. We did this using survey data of respondents from the WEHD 2014 wave. On an individual level, WEHD respondents were classified into high and low exposure to quantitative demands, emotional demands, and low decision authority by classifying approximately 25% of the respondents as truly exposed and 25% as truly unexposed. Job strain was defined as the combination of high quantitative demands and low decision authority by median-split of the continuous scores for the two dimensions. Job insecurity was dichotomized into high ('To a very high extent'/'To a high extent') or low ('To some extent'/'To a low extent'/'To a very low extent'). Work-related physical violence was categorized into yes or no. At the occupational level, individuals from WEHD were then classified into exposed and unexposed based on quartiles of the distribution of each occupational assessed exposure (JEM values) similar to the way it was done in DaWCo. With 2 by 2 tables, sensitivity and specificity were calculated using the formulas and table below:

Sensitivity = $\frac{A}{A+C}$

Specificity = $\frac{D}{B+D}$

	Truly exposed	Truly unexposed
Classified as exposed	A	B
Classified as unexposed	C	D

Using the estimated sensitivity and specificity we produced corrected data from which the bias-adjusted RR for the associations between the six psychosocial working conditions and all length sickness absence (≥1 day) was calculated using the spreadsheet proposed by Lash, Fox, & Fink (6). Sensitivity, specificity, estimated RR, and bias adjusted RR for the six psychosocial working conditions are presented in **table S4** for women and men, separately

Table S4 Sensitivity, specificity, estimated RR, bias adjusted RR for psychosocial working conditions among women and men

	Sensitivity	Specificity	Estimated RR	Bias adjusted RR*
**Women**				
High job insecurity	0.77	0.54	0.96	0.65
High quantitative demands	0.73	0.60	1.14	1.46
Low decision authority	0.69	0.57	1.27	2.38
High job strain	0.66	0.52	1.16	2.23
High emotional demands	0.81	0.63	1.44	2.28
High work-related physical violence	0.98	0.58	1.29	1.98
**Men**				
High job insecurity	0.71	0.53	1.22	-
High quantitative demands	0.74	0.59	0.76	0.19
Low decision authority	0.73	0.59	1.34	2.61
High job strain	0.61	0.51	0.87	-
High emotional demands	0.73	0.60	0.97	0.86
High work-related physical violence	0.94	0.53	1.10	1.90

*Due to low specificity we were unable to estimate bias adjusted RR for men with high job strain and high job insecurity

Appendix 4: Trend over time since labor market entry

Figure S1 distribution of cohabitation

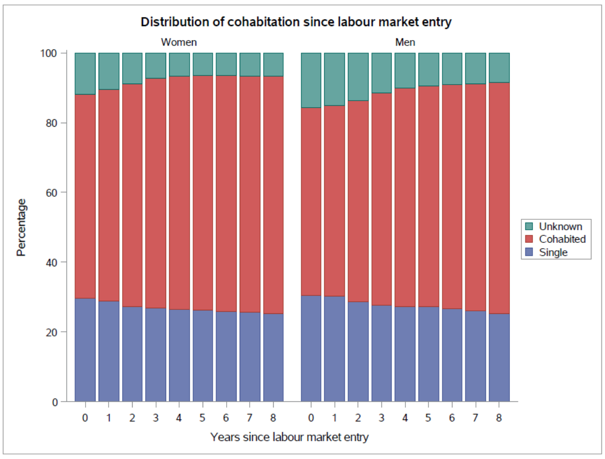

Figure S4 distribution of disposable income

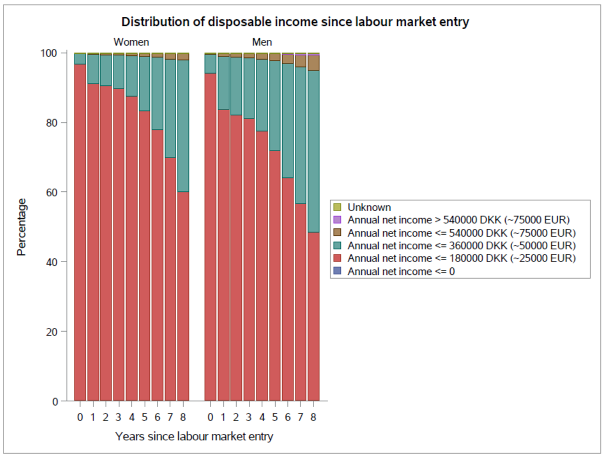

Figure S2 distribution of sector of employment

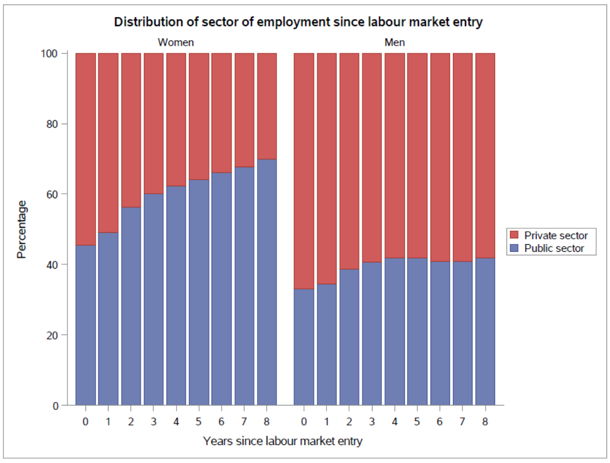

Figure S5 distribution of education

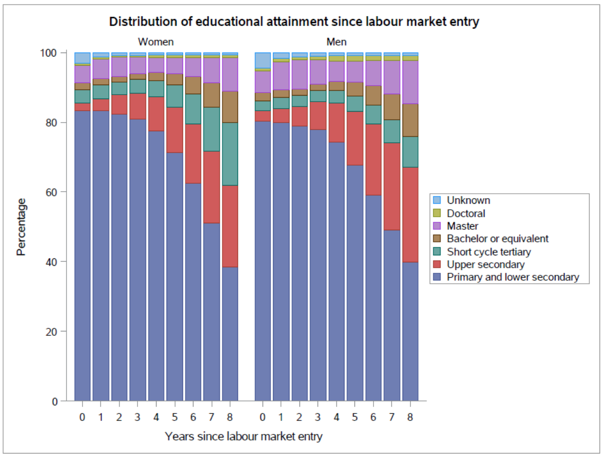

Figure S3 distribution of industry

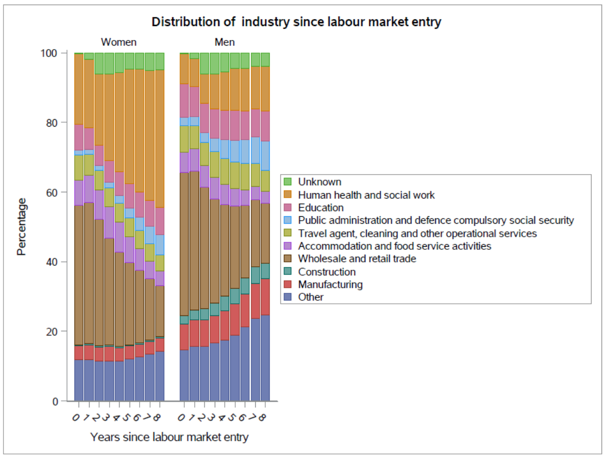

Figure S6 distribution of health service use

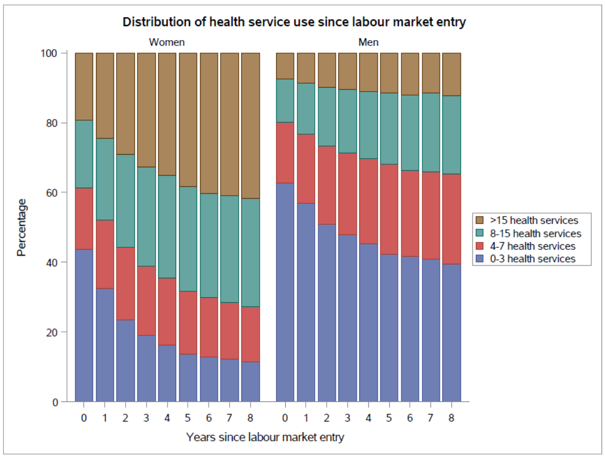

Appendix 5: Step-wise adjustment

Table S6 RR (95% CI) for the stepwise adjusted association between being employed in occupations with specific psychosocial working conditions and SA spells of any length (≥1 day) among women (n = 160,104) and men (n = 141,081)

	Women				Men			
	**Minimal RR (95% CI)**	**Model 2 RR (95% CI)**	**Model 3 RR (95% CI)**	**Fully RR (95% CI)**	**Minimal RR (95% CI)**	**Model 2 RR (95% CI)**	**Model 3 RR (95% CI)**	**Fully RR (95% CI)**
**Job insecurity**								
Low	1.00	1.00	1.00	1.00	1.00	1.00	1.00	1.00
Medium-low	0.80 (0.78 - 0.81)	0.81 (0.80-0.83)	0.82 (0.80-0.83)	0.82 (0.81-0.84)	1.38 (1.35 - 1.41)	1.36 (1.33-1.39)	1.29 (1.27-1.32)	1.26 (1.24-1.29)
Medium-high	1.25 (1.23 - 1.28)	1.25 (1.23-1.27)	1.09 (1.07-1.11)	1.07 (1.05-1.09)	1.29 (1.26 - 1.32)	1.20 (1.18-1.23)	1.15 (1.12-1.17)	1.13 (1.11-1.15)
High	1.07 (1.05 - 1.08)	1.08 (1.06-1.09)	0.97 (0.95-0.99)	0.96 (0.94-0.97)	1.50 (1.46 - 1.53)	1.45 (1.42-1.49)	1.25 (1.22-1.28)	1.22 (1.20-1.25)
**Quantitative demands**								
Low	1.00	1.00	1.00	1.00	1.00	1.00	1.00	1.00
Medium-low	1.18 (1.16 - 1.20)	1.16 (1.14-1.18)	1.19 (1.17-1.21)	1.18 (1.16-1.20)	0.86 (0.84 - 0.88)	0.80 (0.78-0.81)	0.90 (0.89-0.92)	0.92 (0.90-0.93)
Medium-high	1.21 (1.18 - 1.23)	1.18 (1.16-1.20)	1.18 (1.16-1.20)	1.16 (1.14-1.18)	0.90 (0.88 - 0.91)	0.80 (0.79-0.82)	0.87 (0.85-0.88)	0.88 (0.86-0.89)
High	1.05 (1.03 - 1.07)	1.04 (1.02-1.06)	1.15 (1.12-1.17)	1.14 (1.12-1.16)	0.65 (0.64 - 0.67)	0.60 (0.59-0.61)	0.74 (0.72-0.76)	0.76 (0.74-0.78)
**Decision authority**								
High	1.00	1.00	1.00	1.00	1.00	1.00	1.00	1.00
Medium-high	1.46 (1.44 - 1.49)	1.44 (1.42-1.47)	1.29 (1.26-1.31)	1.21 (1.19-1.24)	1.35 (1.32 - 1.38)	1.29 (1.26-1.32)	1.19 (1.16-1.22)	1.17 (1.15-1.20)
Medium-low	1.46 (1.43 - 1.49)	1.44 (1.41-1.47)	1.24 (1.21-1.27)	1.17 (1.15-1.20)	1.44 (1.41 - 1.48)	1.42 (1.39-1.45)	1.24 (1.21-1.27)	1.21 (1.18-1.24)
Low	1.50 (1.47 - 1.53)	1.48 (1.45-1.51)	1.32 (1.29-1.35)	1.27 (1.25-1.30)	1.67 (1.64 - 1.71)	1.72 (1.68-1.76)	1.39 (1.36-1.43)	1.34 (1.31-1.37)
**Job strain**								
Low	1.00	1.00	1.00	1.00	1.00	1.00	1.00	1.00
Medium-low	1.20 (1.18 - 1.22)	1.18 (1.16-1.21)	1.06 (1.04-1.08)	1.03 (1.01-1.05)	0.78 (0.76 - 0.80)	0.77 (0.75-0.79)	0.87 (0.85-0.88)	0.88 (0.86-0.90)
Medium-high	1.46 (1.43 - 1.48)	1.44 (1.41-1.47)	1.30 (1.27-1.32)	1.22 (1.20-1.24)	0.92 (0.91 - 0.94)	0.89 (0.87-0.91)	0.91 (0.89-0.92)	0.92 (0.90-0.94)
High	1.33 (1.30 - 1.35)	1.33 (1.31-1.36)	1.20 (1.18-1.22)	1.16 (1.14-1.18)	0.84 (0.82 - 0.85)	0.80 (0.79-0.82)	0.86 (0.85-0.88)	0.87 (0.86-0.89)
**Emotional demands**								
Low	1.00	1.00	1.00	1.00	1.00	1.00	1.00	1.00
Medium-low	1.08 (1.06 - 1.10)	1.05 (1.03-1.06)	1.07 (1.05-1.09)	1.05 (1.04-1.07)	0.91 (0.89 - 0.93)	0.89 (0.87-0.91)	1.00 (0.98-1.02)	1.00 (0.98-1.02)
Medium-high	1.06 (1.04 - 1.08)	1.09 (1.07-1.11)	1.15 (1.12-1.17)	1.14 (1.11-1.16)	0.81 (0.79 - 0.83)	0.81 (0.80-0.83)	0.92 (0.90-0.94)	0.92 (0.90-0.94)
High	1.64 (1.61 - 1.67)	1.73 (1.70-1.77)	1.54 (1.51-1.57)	1.44 (1.41-1.47)	0.79 (0.77 - 0.81)	0.86 (0.84-0.88)	0.97 (0.95-1.00)	0.97 (0.95-0.99)
**Work-related physical violence**								
Low	1.00	1.00	1.00	1.00	1.00	1.00	1.00	1.00
Medium-low	0.86 (0.84 - 0.87)	0.90 (0.88-0.91)	0.96 (0.94-0.98)	0.97 (0.95-0.99)	1.25 (1.23 - 1.28)	1.20 (1.17-1.22)	1.11 (1.08-1.13)	1.09 (1.07-1.11)
Medium-high	0.88 (0.87 - 0.90)	0.91 (0.90-0.93)	0.92 (0.91-0.94)	0.93 (0.91-0.95)	0.99 (0.97 - 1.01)	0.98 (0.96-1.01)	1.04 (1.02-1.07)	1.04 (1.02-1.06)
High	1.43 (1.41 - 1.46)	1.75 (1.67-1.83)	1.57 (1.49-1.65)	1.41 (1.34-1.49)	1.13 (1.10 - 1.15)	1.23 (1.20-1.25)	1.13 (1.10-1.15)	1.10 (1.08-1.12)

RR: Rate ratio. CI: Confidence intervals: Minimal: Adjusted for age, calendar year, year of labor market entry, years since labor market entry, and years with employment. Model 2: Further adjusted for migration background, cohabitation, and sector of employment. Model 3: Further adjusted for disposable personal income, health services use, existing chronic somatic and mental disorders and physical work demands. Fully: Further adjusted for previous SA.

Appendix 6: Supplementary analyses

Figure S7 RR (95% CI) for the fully adjusted association between being employed in occupations with specific psychosocial working conditions and SA spells of any length (≥ 1 day), SA spells of >1 days, SA spells of >3 days, SA spells of >7 days, and SA spells of >30 days among women (n = 160,104) and men (n = 141,081)

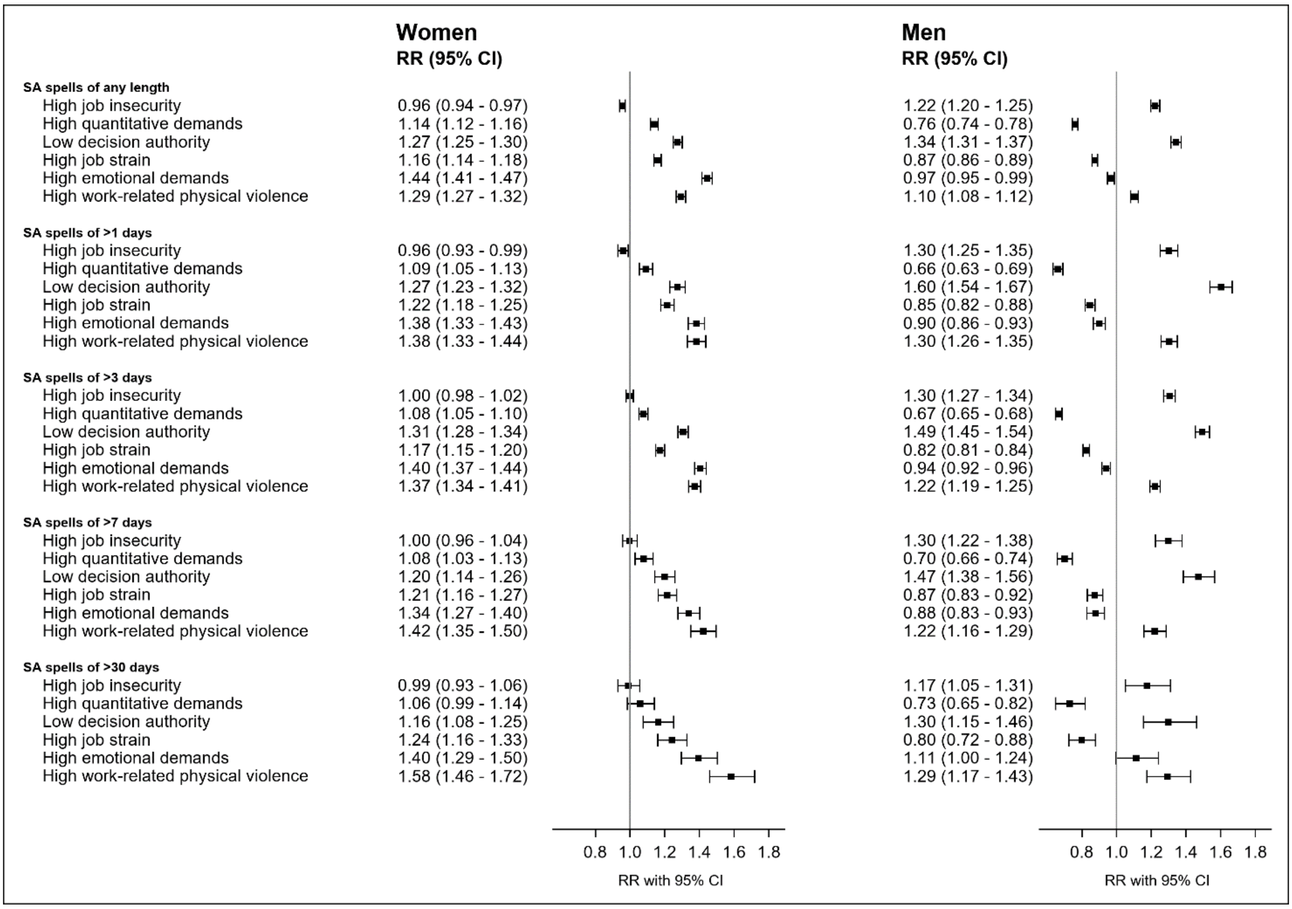

SA: Sickness absence. RR: Rate ratio. CI: Confidence intervals: Adjusted for age, calendar year, year of labor market entry, years since labor market entry, years with employment, migration background, cohabitation, sector of employment, disposable personal income, health services use, existing chronic somatic and mental disorders, physical work demands and previous SA.

Figure S8 RR (95% CI) for the fully adjusted association between being employed in occupations with specific psychosocial working conditions and SA spells of any length (≥ 1 day) stratified by education among women (n = 160,104) and men (n = 141,081)

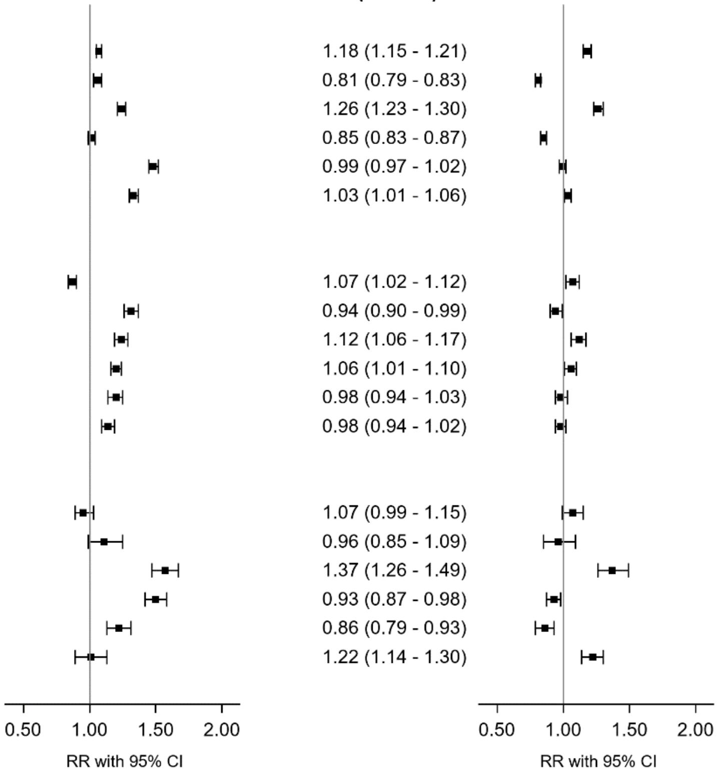

	Women RR (95% CI)	Men RR (95% CI)
**Primary and lower secondary**		
High job insecurity	1.07 (1.05 - 1.09)	1.18 (1.15 - 1.21)
High quantitative demands	1.06 (1.03 - 1.09)	0.81 (0.79 - 0.83)
Low decision authority	1.24 (1.21 - 1.27)	1.26 (1.23 - 1.30)
High job strain	1.01 (0.99 - 1.04)	0.85 (0.83 - 0.87)
High emotional demands	1.48 (1.45 - 1.52)	0.99 (0.97 - 1.02)
High work-related physical violence	1.33 (1.30 - 1.37)	1.03 (1.01 - 1.06)
**Upper secondary & Short cycle tertiary**		
High job insecurity	0.87 (0.84 - 0.90)	1.07 (1.02 - 1.12)
High quantitative demands	1.31 (1.26 - 1.37)	0.94 (0.90 - 0.99)
Low decision authority	1.24 (1.19 - 1.29)	1.12 (1.06 - 1.17)
High job strain	1.20 (1.16 - 1.24)	1.06 (1.01 - 1.10)
High emotional demands	1.20 (1.14 - 1.25)	0.98 (0.94 - 1.03)
High work-related physical violence	1.14 (1.09 - 1.19)	0.98 (0.94 - 1.02)
**Bachelor or equivalent or higher**		
High job insecurity	0.95 (0.89 - 1.03)	1.07 (0.99 - 1.15)
High quantitative demands	1.11 (0.99 - 1.25)	0.96 (0.85 - 1.09)
Low decision authority	1.57 (1.47 - 1.67)	1.37 (1.26 - 1.49)
High job strain	1.50 (1.42 - 1.58)	0.93 (0.87 - 0.98)
High emotional demands	1.22 (1.13 - 1.31)	0.86 (0.79 - 0.93)
High work-related physical violence	1.01 (0.89 - 1.13)	1.22 (1.14 - 1.30)

SA: Sickness absence. RR: Rate ratio. CI: Confidence intervals: Adjusted for age, calendar year, year of labor market entry, years since labor market entry, years with employment, migration background, cohabitation, sector of employment, disposable personal income, health services use, existing chronic somatic and mental disorders, physical work demands and previous SA.

Figure S9 RR (95% CI) for the fully adjusted association between being employed in occupations with specific psychosocial working conditions and SA spells of any length (≥ 1 day) stratified by industry among women (n = 160,104) and men (n = 141,081)

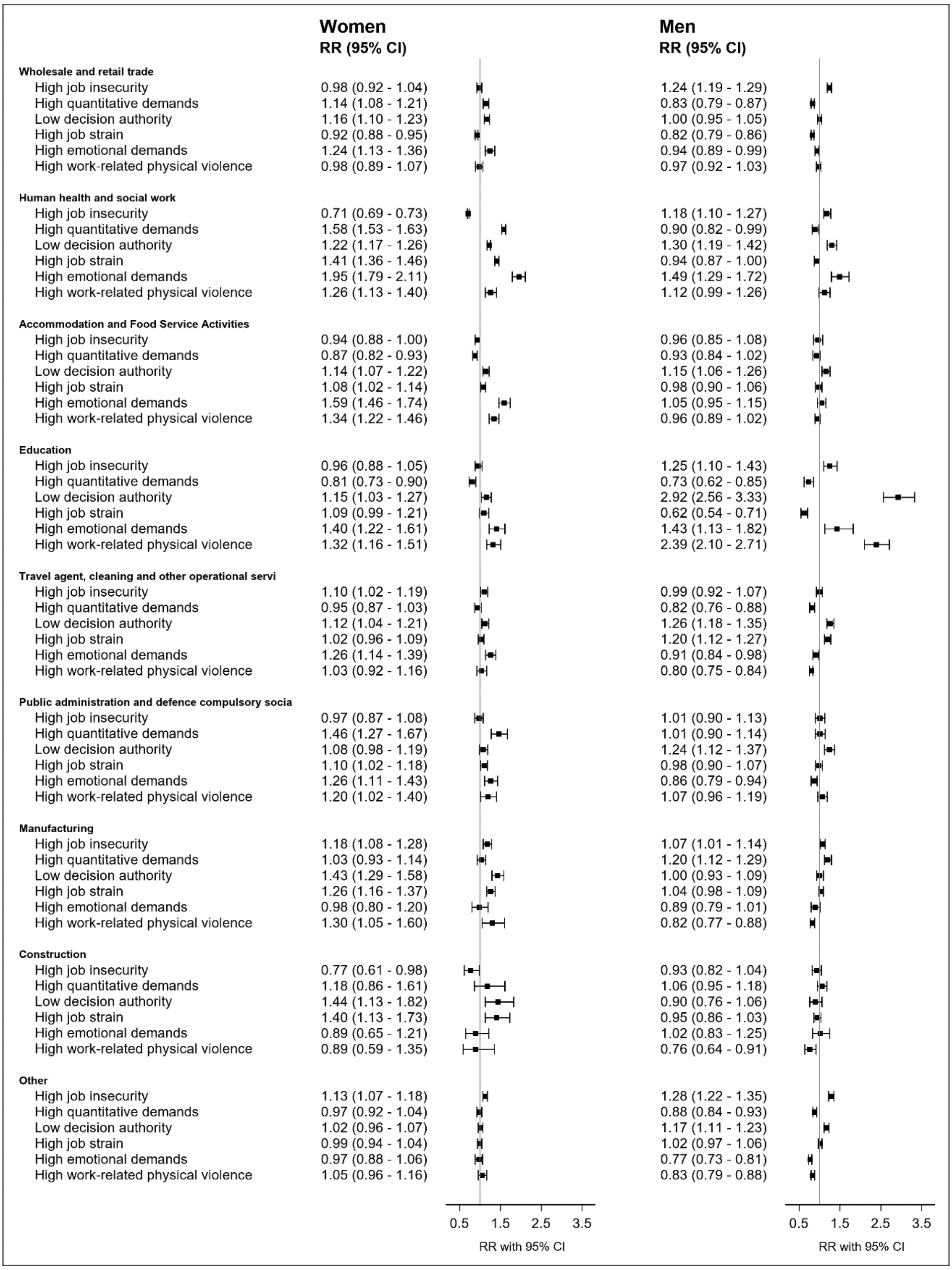

SA: Sickness absence. RR: Rate ratio. CI: Confidence intervals: Adjusted for age, calendar year, year of labor market entry, years since labor market entry, years with employment, migration background,

cohabitation, sector of employment, disposable personal income, health services use, existing chronic somatic and mental disorders, physical work demands and previous SA.

Figure S10 RR (95% CI) for the fully adjusted association between being employed in occupations with specific psychosocial working conditions and SA spells of any length (≥ 1 day) stratified by sector of employment among women (n = 160,104) and men (n = 141,081)

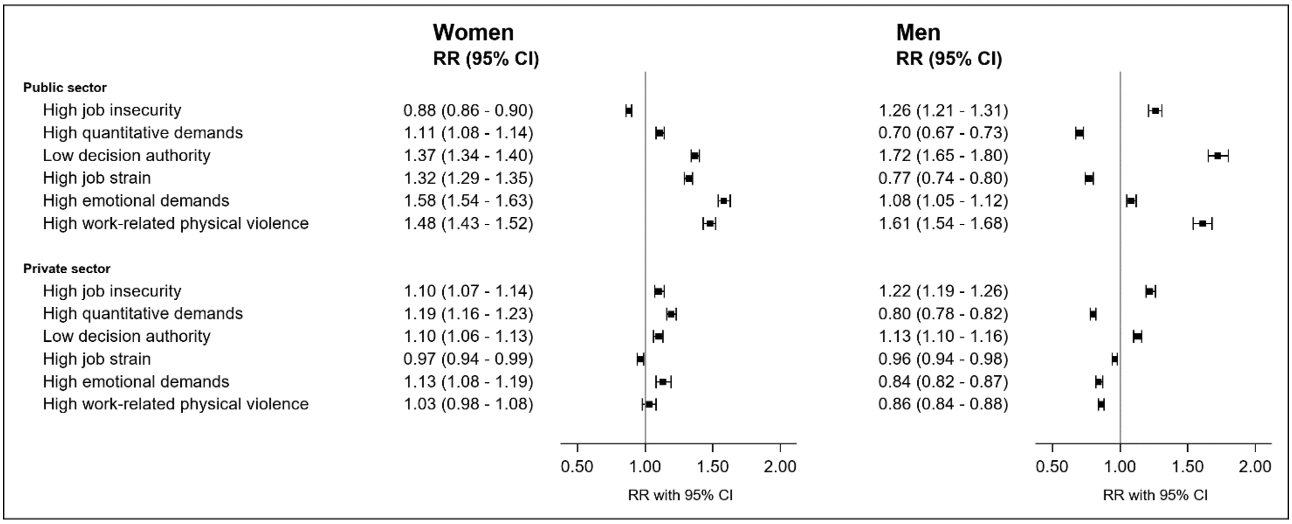

SA: Sickness absence. RR: Rate ratio. CI: Confidence intervals: Adjusted for age, calendar year, year of labor market entry, years since labor market entry, years with employment, migration background, cohabitation, sector of employment, disposable personal income, health services use, existing chronic somatic and mental disorders, physical work demands and previous SA.

Figure S11 RR (95% CI) for the fully adjusted association between being employed in occupations with specific psychosocial working conditions and SA spells of any length (≥ 1 day) stratified by age groups among women (n = 160,104) and men (n = 141,081)

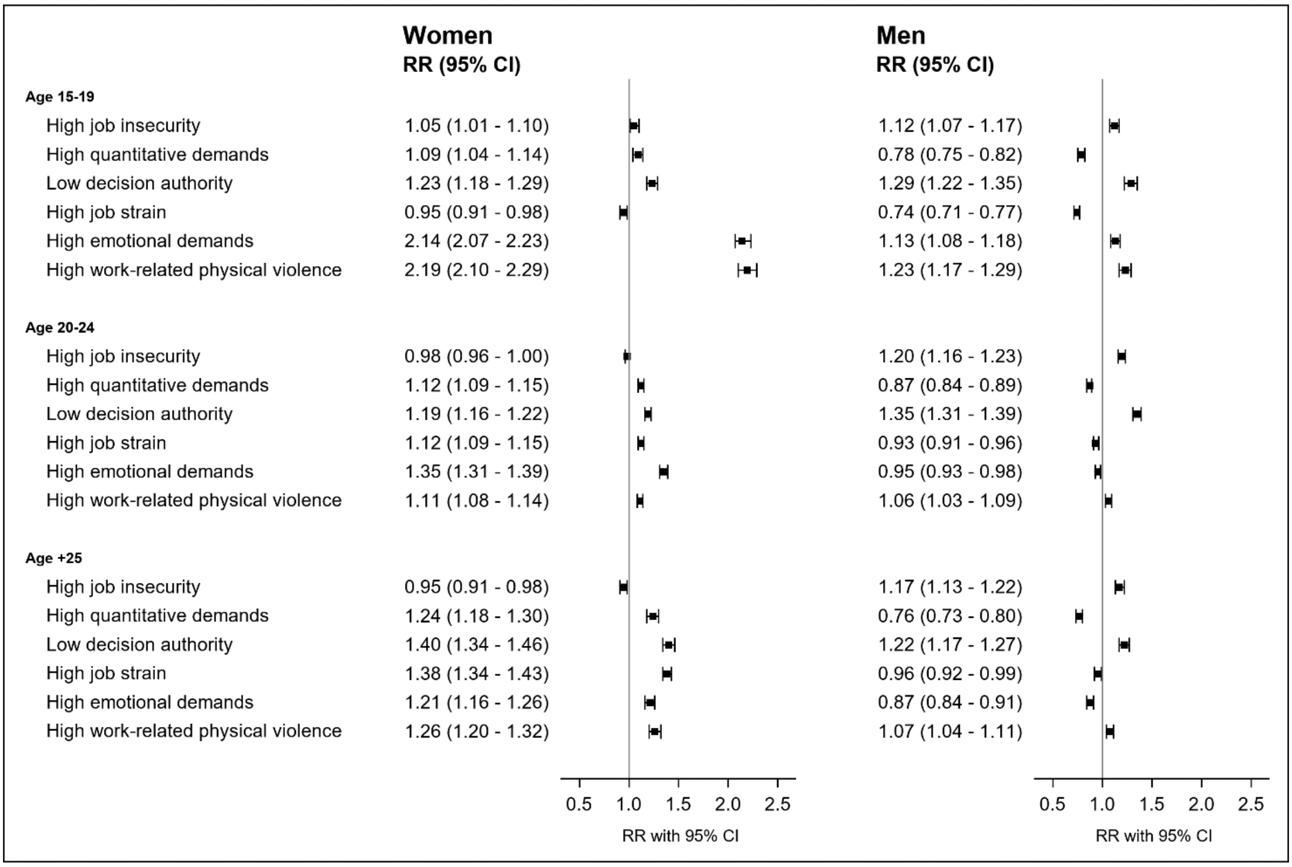

SA: Sickness absence. RR: Rate ratio. CI: Confidence intervals: Adjusted for age, calendar year, year of labor market entry, years since labor market entry, years with employment, migration background, cohabitation, sector of employment, disposable personal income, health services use, existing chronic somatic and mental disorders, physical work demands and previous SA.

Figure S12 RR (95% CI) for the fully adjusted associations between being employed in occupations with specific psychosocial working conditions and SA spells of any length (≥ 1 day) excluding years with non-regular employment and years while under education among women (n = 160,104) and men (n = 141,081)

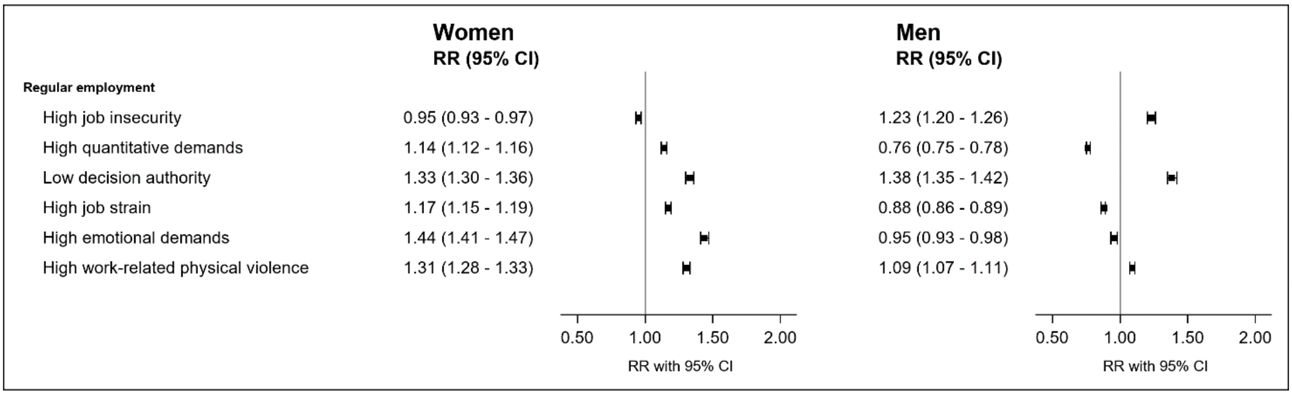

SA: Sickness absence. RR: Rate ratio. CI: Confidence intervals: Adjusted for age, calendar year, year of labor market entry, years since labor market entry, years with employment, migration background, cohabitation, sector of employment, disposable personal income, health services use, existing chronic somatic and mental disorders, physical work demands and previous SA.

References

1. Madsen IEH, Gupta N, Budtz-Jorgensen E, Bonde JP, Framke E, Flachs EM, et al. Physical work demands and psychosocial working conditions as predictors of musculoskeletal pain: a cohort study comparing self-reported and job exposure matrix measurements. Occup Environ Med. 2018;75(10):752-8.
2. Johnsen NF, Thomsen BL, Hansen JV, Christensen BS, Rugulies R, Schlunssen V. Job type and other socio-demographic factors associated with participation in a national, cross-sectional study of Danish employees. BMJ Open. 2019;9(8):e027056.
3. Pedersen CB. The Danish Civil Registration System. Scand J Public Health. 2011;39(7 Suppl):22-5.
4. Thorsen SV, Flyvholm MA, Bultmann U. Self-reported or register-based? A comparison of sickness absence data among 8110 public and private employees in Denmark. Scand J Work Environ Health. 2018;44(6):631-8.
5. Thygesen LC, Daasnes C, Thaulow I, Bronnum-Hansen H. Introduction to Danish (nationwide) registers on health and social issues: structure, access, legislation, and archiving. Scand J Public Health. 2011;39(7 Suppl):12-6.
6. Lash TL, Fox MP, Fink AK. Applying quantitative bias analysis to epidemiologic data: Springer; 2009.
